# Genetic diversity and connectivity of chemosynthetic cold seep mussels from the U.S. Atlantic margin

**DOI:** 10.1186/s12862-022-02027-4

**Published:** 2022-06-17

**Authors:** Danielle M. DeLeo, Cheryl L. Morrison, Makiri Sei, Veronica Salamone, Amanda W. J. Demopoulos, Andrea M. Quattrini

**Affiliations:** 1 Department of Invertebrate Zoology, National Museum of Natural History, Smithsonian Institution, 10th and Constitution Ave NW, Washington, DC 20560 USA; 2 U.S. Geological Survey, Eastern Ecological Science Center, 11649 Leetown Road, Kearneysville, WV 25430 USA; 3 US Geological Survey, Wetland and Aquatic Research Center, 7920 NW 71st St, Gainesville, FL 32653 USA

**Keywords:** RADseq, SNP, Genomics, Adaptation, Chemosynthetic, Bathymodioline, *Gigantidas childressi*, *Bathymodiolus heckerae*, Recruitment

## Abstract

**Background:**

Deep-sea mussels in the subfamily Bathymodiolinae have unique adaptations to colonize hydrothermal-vent and cold-seep environments throughout the world ocean. These invertebrates function as important ecosystem engineers, creating heterogeneous habitat and promoting biodiversity in the deep sea. Despite their ecological significance, efforts to assess the diversity and connectivity of this group are extremely limited. Here, we present the first genomic-scale diversity assessments of the recently discovered bathymodioline cold-seep communities along the U.S. Atlantic margin, dominated by *Gigantidas childressi* and *Bathymodiolus heckerae*.

**Results:**

A Restriction-site Associated DNA Sequencing (RADSeq) approach was used on 177 bathymodiolines to examine genetic diversity and population structure within and between seep sites. Assessments of genetic differentiation using single-nucleotide polymorphism (SNP) data revealed high gene flow among sites, with the shallower and more northern sites serving as source populations for deeper occurring *G. childressi*. No evidence was found for genetic diversification across depth in *G. childressi*, likely due to their high dispersal capabilities. Kinship analyses indicated a high degree of relatedness among individuals, and at least 10–20% of local recruits within a particular site. We also discovered candidate adaptive loci in *G. childressi* and *B. heckerae* that suggest differences in developmental processes and depth-related and metabolic adaptations to chemosynthetic environments.

**Conclusions:**

These results highlight putative source communities for an important ecosystem engineer in the deep sea that may be considered in future conservation efforts. Our results also provide clues into species-specific adaptations that enable survival and potential speciation within chemosynthetic ecosystems.

**Supplementary Information:**

The online version contains supplementary material available at 10.1186/s12862-022-02027-4.

## Background

Bathymodioline mussels that dominate many cold-seep communities serve as characteristic indicator fauna for methane and sulfide rich environments (reviewed in [1]). They thrive in both cold-seep and hydrothermal-vent environments [2], largely due to a crucial symbiosis with chemoautotrophic and/or methanotrophic bacteria that oxidize sulfur and/or methane for nutrition [1, 3, 4]. Bathymodiolines function as ecosystem engineers, forming large mussel bed aggregations on the benthos that serve as important foundation habitat for other seep-adapted fauna [5], including a variety of endemic species [6]. Further characterization of the population structure, dynamics and connectivity for this ecologically important group of deep-sea species is timely as resource (e.g., mineral, oil and gas) extraction efforts continue in deep waters and may impact these habitats.

**Fig. 1:**
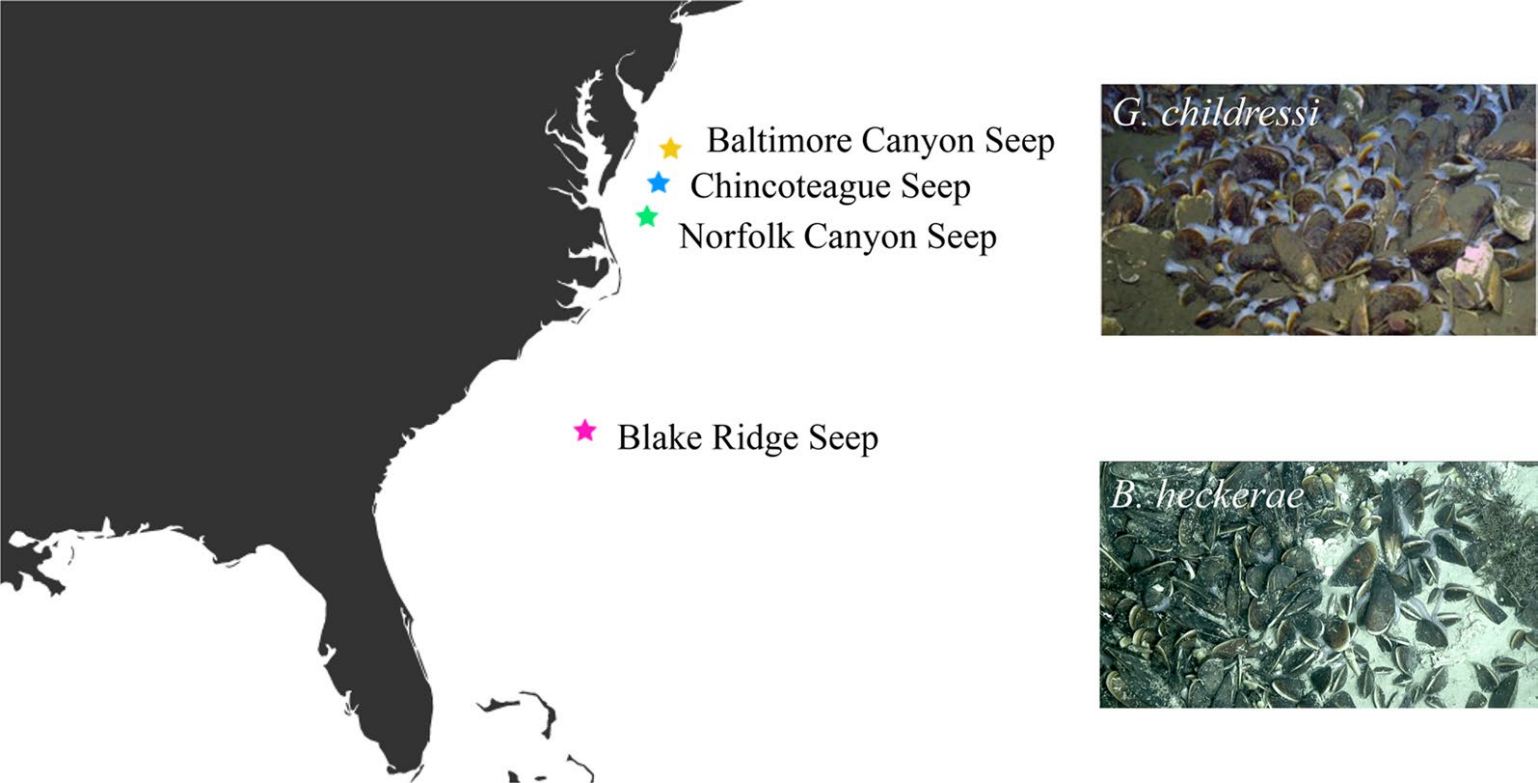
Map of sampling locations along the U.S. Atlantic Margin. Norfolk Canyon Seep= NCS, Baltimore Canyon Seep = BCS, Chincoteague Seep = CTS. Photo credit: DeepSearch

Chemosynthetic cold seeps are numerous along the U.S. Atlantic margin [7], though they remain poorly characterized. Widespread methane leakage with > 500 gas plumes have been noted from Cape Hatteras to Georges Bank [7], however, there have been few ground-truthing surveys and only a handful of bathymodioline mussel beds have been documented [7–9]. Observations of mussel beds along the margin include one of the first seeps discovered in the region in deep waters (~ 2100 m) on the Blake Ridge diaper off South Carolina [10, 11] and among submarine canyons off the coast of Baltimore, Maryland and Norfolk, Virginia [7–9] (Fig. 1). Understanding the dynamics of these foundation species, their influence on nearby and/or distant communities and the factors that impact larval dispersal, settlement and survival is crucial for informing future exploration and management efforts, given seep habitats are still being discovered.

Previous investigations of cold-seep communities along the Atlantic Equatorial Belt, from the Gulf of Mexico to the Gulf of Guinea, found that depth, rather than geographic distance, was the primary driver structuring communities [11]. Evidence for segregation with depth also exists among bathymodioline species commonly found in the U.S. Atlantic. For example, *Gigantidas childressi* [12] (*= Bathymodiolus childresssi*), which dominates the known seep sites within the aforementioned submarine canyons [13], is typically found between 400 and 2200 m, while *Bathymodiolus heckerae* [12] is the dominant species found among deeper cold seeps between 2200 and 3300 m [13], including those found at Blake Ridge [11, 14]. As several bathymodioline mussel species are believed to be panmictic [15–17] with long-range dispersal capabilities [18], underlying genetic differences may be influencing the fitness of one or both species and their ability to colonize and thrive at different depths. Of particular note, bathymodiolines are thought to host different chemosynthetic endosymbiotic bacteria (e.g. [19]) that may enable optimal utilization and turnover of chemical nutrients for energy. In addition to nutrient type and availability, depth segregation [11] suggests adaptations among species inhabiting specific depth niches. Therefore, genetic differences that are influenced by environmental changes associated with depth (e.g., temperature and pressure) and/or the chemical composition of seep fluids likely exist among bathymodiolines. With the continual optimization of high-throughput sequencing approaches for non-model organisms, genome-wide diversity assessments could clarify how selective pressures have influenced diversification within this group.

Most previous studies focusing on population connectivity and genetic diversity in bathymodiolines have used markers that were perhaps not informative enough (see [17]) or not neutrally-evolving (e.g., strong or relaxed purifying selection in several mitochondrial genes, [19]), thereby hindering accurate inference of gene flow, population differentiation, and genomic diversity [20, 21]. Although prior studies have provided important insights on the evolution within this genus, these studies along with others that used a handful of more variable markers (i.e., microsatellites) often concluded regional panmixia within a species across 10 to 100s of km and 100s of m of depth (e.g., [16, 17, 22]). These findings raise the question of whether the markers used were informative enough to detect fine-scale population structure, if it exists.

While there are many methods to ascertain genome-wide informative markers, Restriction-Site Associated DNA Sequencing (RADSeq) has emerged as a popular method for marker discovery in population genomics [23–25]. RADSeq relies on restriction enzymes and high-throughput sequencing to produce 1000 s of genomic markers, from which SNPs can be attained and subsequently used in downstream analyses. SNPs can be used to determine levels of genetic diversity and rates of gene flow and to discover genes that are potentially indicative of adaptation to environmental conditions.

Here, we present a genome-wide diversity and connectivity assessment of the recently discovered bathymodioline cold-seep communities along the U.S. Atlantic margin, dominated by *G*. *childressi* along the upper to middle continental slope and *B. heckerae* in lower-slope depths at Blake Ridge. Due to the potentially large dispersal range of these mussels, we hypothesized that the discrete communities of *G. childressi* would show minimal levels of differentiation that would likely increase across depth, with the highest levels of divergence between the shallowest (Baltimore Canyon) and deepest (Norfolk Canyon) seeps. We also examined whether genes potentially under selection in bathymodiolines would be related to the different depths that they inhabit, thus providing further insight into molecular underpinnings of niche ranges in cold-seep mussels.

## Methods

### Sample collections and extraction

*G. childressi* were collected from Norfolk Canyon Seep (NCS; 1485–1600 m depth), Baltimore Canyon Seep (BCS; 360–430 m depth) and Chincoteague Seep (CTS; 1040 m) (Fig. 1) using a scooping method from a remotely operated vehicle, with scoops of mussels placed in a lidded-collection container on the working basket of the vehicle (see [12, 26] for further information). The vast majority of *B. heckerae* samples were collected from Blake Ridge Seep (BRS) using mussel ‘pots’ (lined pots placed in quivers- i.e., plastic buckets) from either the human-operated vehicle (HOV) *Alvin* or the remotely-operated vehicle (ROV) *Jason II* (dives A4967 and J2-1136) with the exception of two individuals. These two samples were collected via (1) slurp, a vacuum-like device with an enclosed system where the sample is housed for the duration of the dive (1: dive J2-1136, BRS) and (2) by a ROV arm grab (2: dive HRS1704-GEX03-023, from a shallower region near NCS). Tissues were preserved in 100% ethanol and kept at − 20 °C until they were processed. Mantle or foot tissues were ultimately used for total genomic DNA extractions to limit DNA contamination by symbionts hosted in the gills using either an AutoGenPrep 965 Kit (Autogen, Holliston, MA, USA), the E.Z.N.A.^®^ Mollusc DNA Kit (Omega Bio-Tek, Norcross, GA, USA) for smaller tissue-limited samples, or MagAttract HMW DNA Kit (Qiagen, Hilden, Germany) for low yield samples.

### PCR and sanger sequencings

Species identifications were confirmed using morphology (as in [27]) and mitochondrial COI barcodes. *G. childressi* individuals were confirmed in a previous study [12], and representative COI sequences (NCBI PopSet: 1485777102) from that study were compared to examine overall sequence divergence. To generate *B. heckerae* cytochrome c oxidase subunit 1 (COI) barcodes, PCR was conducted in 10 µl volumes (3.2 µl nuclease-free water, 0.1 µl BSA, 0.1 µl DMSO, 0.3 µl forward primer, 0.3 µl reverse primer and 5.0 µl GoTaq® Hot Start Master Mix (Promega, Madison, WI, USA) using the following primers: jgLCOI (TITCIACIAAYCAYAARGAYATTGG) and jgHCOI (TAIACYTCIGGRTGICCRAARAAYCA) [28]. The targeted COI region was amplified with the following protocol: initial denaturation at 95 °C for 7 min, followed by 40 cycles of (denaturation at 95 °C for 45 s, annealing at 42 °C for 45 s, extension at 72 °C for 60 s), and a final extension of 72 °C for 5 min. Bidirectional Sanger sequencing was performed at the National Museum of Natural History’s Laboratories of Analytical Biology using ABI 3730xl Genetic Analyzer (Thermo Fisher, Waltham, MA, USA). Raw chromatograms were assembled into bidirectional consensus sequences and edited using Geneious Prime 2020.1.2 [29] (https://www.geneious.com). For both species, COI sequence divergence (i.e., 100% identity) was estimated in Geneious following sequence alignment with MUSCLE using default parameters.

### RAD-sequencing

*Bathymodiolus heckerae* and *G. childressi* genomic (g)DNA were quantified with the Qubit dsDNA BR Assay Kit (Thermo Fisher), and the presence of high molecular weight DNA was confirmed with 0.8% agarose gel electrophoresis. The gDNA was normalized to 20 ng/µl in 50 µl volumes and sent to Floragenex (Beaverton, OR). DNA libraries were constructed using the 6-cutter *PstI* enzyme, followed by sequencing 100 bp single-end reads across four lanes of an Illumina HiSeq2500 (University of Oregon’s Genomics and Cell Characterization Core Facility lab). In total 183 bathymodiolines were sequenced, 86 *G. childressi* and 97 *B. heckerae* samples (Additional file 1: Table S1).

### Population genomics

Raw data were quality checked using the program FASTQC [30] and de-muliplexed using the program STACKS (*process_radtags*, -e pstI --inline_null) with default parameters to clean the data (-c: removing any read with an uncalled base and -q: discarding reads with low quality scores) [31]. RADseq data for each species was assembled discretely with iPYRAD v 0.9.12 [32] using the genome of *Gigantidas platifrons* [33] as reference [34] (NCBI accession GCA_002080005.1). As there is no available genome for either species, the only available genome of a bathymodioline relative was used to guide the assembly of the RADseq data based on the recommendations of [35]. Reference-based assembly methods assist the assembly process by removing/masking repetitive regions and paralogous sequences and leads to a higher number of SNPs, reduces *F*_IS_ and transition to transversion ratios, and produces more accurate summary statistics as compared to *de novo* approaches [35]. The referenced based assemblies were done using default parameters, with a sequence similarity (*clust_threshold*) of 0.95, maximum missing data of 20% (*min_samples_locus =* 61 and 72 for *G. childressi* and *B. heckerae* respectively), and a maximum number of heterozygous sites (*max_shared_Hs_locus*) of 0.5. To maximize the loci (gene or allele position) recovered, individuals with < 1000 k assembled contigs (*G. childressi* n = 6 and *B. heckerae* n = 9) were excluded from the analyses. Following assembly, loci and corresponding SNP information were recovered for each species (Table 1).

Additional downstream analyses were run in iPYRAD using jupyter notebook according to the API: iPYRAD assembly workflow [36]. To examine genetic population structure between the seep sites for *G. childressi*, unlinked SNPs (n = 21,220) were used in the program STRUCTURE v.2.3.4 via the iPYRAD-analysis toolkit, as described in the workflow. STRUCTURE [37] estimates admixture (i.e., hybridization) and population structure between populations (sites) using multi-locus genotypes (e.g., SNPs). Population information was assigned based on seep locality (group = Norfolk, Baltimore or Chincoteague) for *G. childressi*, requiring 50% SNP coverage in each group defined above (*minmap* = 0.5). SNPs were further filtered by removing those not shared across 90% of all samples (*mincov* = 0.9). STRUCTURE was run in replicate (n = 10, *burnin =* 20,000, *numreps =* 100,000) using several population (K) values (K = 1 – 4). The rate of change in log probabilities (deltaK) and mean log probabilities were plotted in the package toyplot to determine the most likely number of (K) populations [38]. Probability of membership of each individual to K clusters were averaged across 10 iterations and also plotted in toyplot.

Further assessment of genetic differentiation among *G. childressi* and *B. heckerae* was done in R (v.3.5.0) using the package *adegenet* [39]. For *G. childressi*, site information was added as a hypothetical population factor (genind@pop) to examine genetic diversity among the northernmost seep sites (NCS, BCS, CTS; Fig. 1). As almost all *B. heckerae* samples (except one individual) were sampled from the same locality (BRS), sampling methods were added to the analysis to examine the influence of sampling methodology (ROV, slurp or Mussel pot) on genetic diversity estimates. For *G. childressi*, loci were further filtered for missing data in R using *poppr* v2.8.5 [40] to remove loci with any missing data across individuals (*missingno*, type = “loci”, cutoff = 0). This subset (n = 16,720) was further filtered to remove non-neutral loci (p < 0.05), potentially under selection, based on Hardy-Weinberg Equilibrium (HWE) tests (hw.test) conducted in R using *pegas* [41] resulting in 11,995 loci. Due to a lower yield of assembled loci for *B. heckerae* (~ 0.2X), no further filtering was conducted on the loci to maximize data available for downstream analyses. Genetic diversity statistics were computed using the *basic.stats* function from the package *hierfstat* [42], including the population differentiation fixation index (*F*, inbreeding coefficient (*F*_IS_), mean observed heterozygosity (*H*_*O*_), mean gene diversities within populations (*H*_*S*_), mean gene diversity overall (*H*_*T*_), all calculated according to Nei [43]. Additionally, a measure of population differentiation (*D*_*EST*_) as in Jost [44] was calculated. Pairwise *F*_ST_ comparisons were also computed between the cold-seep localities for *G. childressi*. A Principal Components Analysis (PCA) was performed in R on both the putatively neutral *G. childressi* subset (n = 11,995 loci) and *B. heckerae* assembly using *ade4* [45]. Missing values were replaced by the mean allele frequencies (*NA.method *= "mean”).

**Table 1:** Overview of the final, filtered RADseq assemblies, subsequent processing and corresponding analyses performed for *G. childressi* and *B. heckerae*

Species	Assembly	# Loci	# SNPs	Analysis
*G. childressi*	Final	21,292	283,754^b^	SEQUOIA (relatedness)
			21,220 (unlinked)	STRUCTURE (population structure/admixture)
	Excluding missing data^a^	16,720	39,551	BA3 (intra-site inbreeding), HWE tests (neutrality)
		11,995 (neutral)	1762 (unlinked)	Genetic diversity estimates, PCA, BA3 (inter-site migration)
*B. heckerae*	Final	4142	52,904^b^	SEQUOIA
			4114 (unlinked)	Genetic diversity estimates, PCA
Combined bathymodioline reference	Final	94,867	791,594	pcadapt (outlier SNPs), REVIGO

^a^No loci with any missing data included

^b^Total SNPs recovered by iPYRAD for each species prior to additional downstream filtering (if any)

### Connectivity and gene flow

To assess relatedness among (*G. childressi*) and within (*G. childressi* and *B. heckerae*) seep communities, kinship analyses were conducted in SEQUOIA [46] which performs pedigree reconstruction based on SNP data. The vcf file from iPYRAD was converted into a raw readable format using PLINK (v1.90b6.21,http://pngu.mgh.harvard.edu/purcell/plink/ [47]). The SNPs were then randomly subset with PLINK following recommendations in [46], with parameter thresholds for missingness (*--geno* 0.2), minor allele frequency (*--maf* 0.3) and the sliding window (*--indep* 50 5 1). The SNP subsets (*G. childressi* n = 669 and *B. heckerae* n = 628) were then extracted using PLINK (--*extract* --*recodeA*) and converted into genotype data in SEQUOIA (*GenoConvert*) to use as input for the pedigree reconstruction (function *sequoia, MaxSibIter* = 40, *Err* = 0.001, *FindMaybeRel* = TRUE). For each pair of individuals, likelihoods were calculated to determine parent-offspring (PO), full siblings (FS), half siblings (HS), grandparents (GP), full avuncular (niece/nephew - aunt/uncle; FA), half avuncular/ great-grandparental/cousins (HA), or unrelated (U) relationships. Kinship assignments were made if the log likelihood ratio (LLR) between a given relationship and the most likely alternative exceeded the default threshold.

To further assess contemporary connectivity and the directionality of gene flow among *G. childressi* communities, recent migration rates were calculated between the three sampling localities (NCS, CTS, BCS) in BayesAss v3.04 (BA3) [48]. BA3 assumes that first generation immigrants can be detected and mean immigration rates for each population can be estimated. Migration rates were calculated using only neutral loci and unlinked SNPs. First, the locus subset filtered for missing data (n = 16,720 loci) was used to calculate site-specific inbreeding coefficients with BA3 using 10,000,000 iterations, with a burnin of 250,000 and sampling frequency every 100 generations. Parameters were set to ensure acceptance rates between 20 and 60% for all adjustable parameters (according to [48]), with allele (-a) frequencies at 0.3, inbreeding coefficients (-f) at 0.02, and migration rates (-m) at 0.1 (default). Then to calculate migration rates, the neutral locus dataset (n = 11,995 loci) was then re-run in BA3 using the same run settings, but with parameters slightly adjusted to ensure acceptance rates remained within the appropriate range (a = 0.35, f = 0.02 and m = 0.15). In both scenarios, convergence was examined and confirmed using Tracer v1.7.1 [49].

### Signature of selection by depth

To examine loci under potential selection in each species that inhabit distinct depth ranges (*G. childressi* - shallower seeps, 400–2200 m vs. *B. heckerae* - deeper seeps, 2200 to 3300 m; [50]), a subset of sequence data that maximized the quality and size of locus yield were chosen for analyses (Table 1). Twenty-eight individuals were chosen based on the number of loci assembled in each species-specific assembly (*G. childressi* > 28 K loci with spread across all three sampling localities (NCS = 14, BTC = 10, CTS = 4); *B. heckerae* > 3.8 K loci). The 54 individuals were re-run in iPYRAD [32] with the reference genome of *G. platifrons*, with default parameters described above (*clust_threshold* = 0.85, *min_samples_locus =* 45, *max_shared_Hs_locus* = 0.25). Genotype data were extracted from the vcf file and converted to bed format using PLINK. These data were then analyzed in R using *pcadapt* [51] following the *pcadapt* vignette (https://github.com/bcm-uga/pcadapt/blob/master/vignettes/pcadapt.Rmd) to detect genetic markers potentially under selection (outlier SNPs) using Principal Components Analysis (PCA). Following [51], the PCA was first performed with a large number of principal components (K = 20) and examined to assess the percentage of variance explained by each PC. This was visualized to assess the PC/eigenvalues that correspond to population structure (steep curve, left of straight line) vs. random variation (straight line). In addition, a score plot was generated to visualize population structure (or in this case species structure) of the samples according to the principal components (PC). The optimal number of PCs was determined from these plots (K = 2) and pcadapt was re-run accordingly with default parameters. Test statistics for each SNP based on the PCA and outliers based on Mahalanobis distance, a multidimensional approach to measure distance from the mean [51], were estimated using *pcadapt*. A Bonferroni correction was applied and SNPs were considered outliers based on the adjusted p-values (≤ 0.05).

Corresponding loci and genomic annotations from the published *G. platifrons* genome were identified for the outlier SNPs via custom scripts (see https://github.com/deleod) using biopython [52]. Outlier SNPs were linked back to the *G. platifrons* scaffolds with annotation information from the *G. platifrons* genome [34] downloaded from NCBI (BioProject PRJNA328542, accession MJUT00000000). BEDTools [53] was then used to extract gene information from the corresponding genome GFF annotation file, which has gene ontology (GO) and KEGG orthology (KO) information. REVIGO (http://revigo.irb.hr/, [54]) was then used to produce a reduced visualization of the biological processes and molecular functions associated with these outlier SNPs, indicative of select loci under putative selection. REVIGO summarizes GO information by reducing functional redundancies based on semantic similarity (most informative common term). KEGG (Kyoto Encyclopedia of Genes and Genomes) reconstruction pathway mapper [55] was further used to elucidate corresponding pathways associated with the outlier SNPs. KEGG is a collection of databases corresponding to biological pathways in addition to genomic, disease and chemical substance information. The KEGG Reconstruct tool can be used to “reconstruct” biological pathway maps from a set of K numbers (KO identifiers or annotation IDs) obtained from the annotated genome of *G. platifrons*.

## Results

An average of 96.2% of reads were retained after demultiplexing and filtering with *STACKS.* This includes 4.27 M (± 2.18 M) reads for *G. childressi* and 4.18 M (± 3.63 M) for *B. heckerae.* DNA barcoding of the COI gene indicated pairwise sequence divergences ranging from 0 to 2.12% for *G. childressi* and 0 to 1.22% for *B. heckerae*.

### Genetic connectivity and diversity

#### *Gigantidas childressi*

On average ~ 99.9% of reads for *G. childressi* passed the additional filtering step in iPYRAD, which generated an average of ~ 271.4 K total clusters per individual with an average heterozygosity estimate of 0.01 and error estimate of 0.002. A total of 417,015 loci were assembled using the reference genome of *G. platifrons*. Loci retained following filtering for a maximum of 20% missing individuals per locus, resulted in 21,292 loci and 283,754 total SNPs (39.0% missing sites and 21,220 unlinked SNPs).

Assessments of genetic differentiation among *G. childressi* yielded an overall *F*_ST_ of 0.002 with a low overall mean observed heterozygosity (*H*_*O*_ = 0.09), within population gene diversity (*H*_*S*_ = 0.13) and overall gene diversity (*H*_*T*_ = 0.13) (Table 2). These results indicated low heterozygosity and relatively equal allele frequencies among *G. childressi* individuals collected from the different seep sites along the Mid-Atlantic margin. Further, *G. childressi* had an overall inbreeding coefficient (*F*_IS_) of approximately 0.32, indicating a high level of inbreeding among the total population of sampled individuals in this region. However, inbreeding coefficients were generally lower at each site (*F*_*IS*_= 0.04–0.10) (Table 3).

**Table 2:** Overall summary statistics across neutral loci calculated in *hierfstat*

Species	*N*	*H*_*O*_	*H*_*S*_	*H*_*T*_	*D*_*ST*_	*F*_*ST*_	*F*_*IS*_	*D*_*EST*_
*G. childressi*	81	0.0860	0.1276	0.1278	0.0003	0.0022	0.3260	0.0005
*B. heckerae*	87	0.0743	0.1064	0.1064	0.0000	0.0000	0.3015	–

*N*: number of individuals, *F*: Fixation Index, *H*_*O*_: mean observed heterozygosity, *H*_*S*_: within population expected heterozygosity, *H*_*T*_: overall gene diversity, *D*_*ST*_: amount of gene diversity among samples (*H*_*T*_-*H*_*S*_), *F*_*IS*_: inbreeding coefficient, *D*_*EST*_: measure of population differentiation

**Table 3:** BayesAss estimated inbreeding coefficients (*F*_*IS*_) at each site with standard error (S.E.)

Site	Inbreeding coefficient (S.E.)
NCS	0.041 (0.065)
CTS	0.041 (0.048)
BCS	0.104 (0.021)

Pairwise *F*_*ST*_ values between the collection localities were low and ranged from 0.003 to 0.006 (Table 4). The highest pairwise *F*_*ST*_ value was between Baltimore Canyon Seep (BCS) and Chincoteague Seep (CTS), followed by Norfolk Canyon Seep (NCS) and CTS, indicating relatively larger genetic differences between the canyon seeps and CTS. This is despite Chincoteague’s relatively intermediate location and depth, though this may be due to the low number of samples available for this locality (n = 6).

**Table 4:** Pairwise *F*_*ST*_ values for *G. childressi* collected from three cold seep communities along the Mid-Atlantic margin

Site	NCS n = 53	CTS n = 6	BCS n = 22
NCS	–	–	–
CTS	0.005	–	–
BCS	0.003	0.006	–

Norfolk Canyon Seep (NCS, 1485–1600 m), Chincoteague Seep (CTS, 1037 m) and Baltimore Canyon Seep (BCS, 360–430 m). Values represent putative population differentiation based on genetic structure

**Fig. 2:**
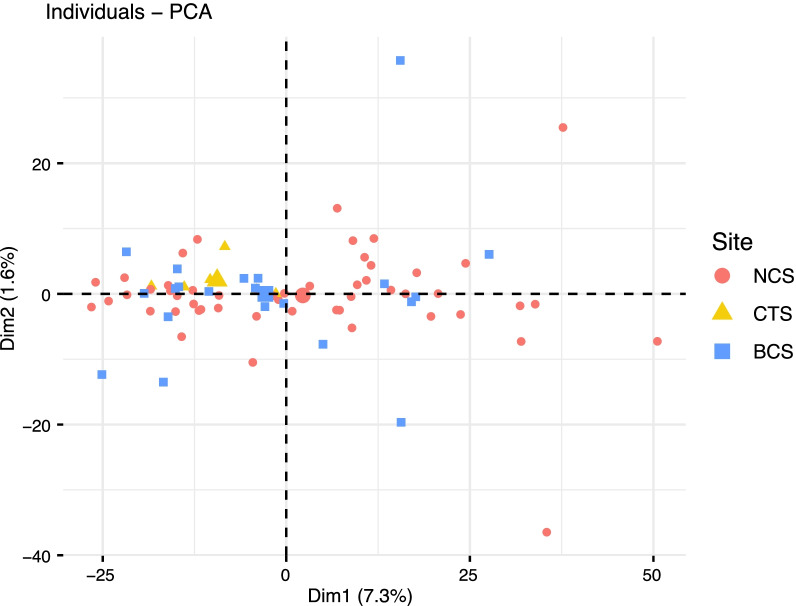
Principal components analysis (PCA) plot representing genetic differentiation among *G. childressi* samples collected from Norfolk Canyon Seep (NCS, red circles), Chincoteague Seep (CTS, orange triangles) and Baltimore Canyon Seep (BCS, blue squares)

**Fig. 3:**
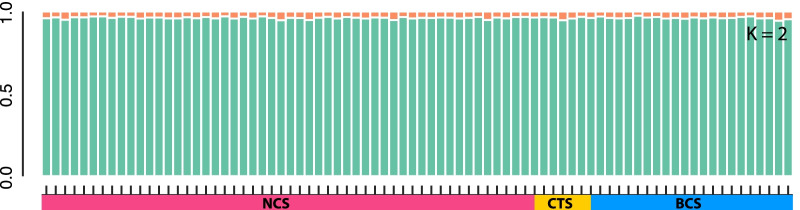
Average probability of membership graph for *G. childressi* (n = 81) collected from seeps at Norfolk canyon (NCS), Chincoteague (CTS) and Baltimore Canyon (BCS). K = 2 clusters (or ancestral populations) as identified by STRUCTURE

Principal Components Analysis (PCA) used to visualize genetic clustering among *G. childressi* individuals and across collection localities also showed a considerable amount of overlap among samples (1: NCS [n = 53], 2: CTS [n = 6], 3: BCS [n = 22], Fig. 2). The first PCA axis explained 7.3% of variation while the second axis explained 1.6%. Genetic similarity among sites was also apparent by the lack of clustering among individuals collected from the same seep community. Similarly, STRUCTURE analyses indicated no population structure among *G. childressi* from the three seeps. Plots based on both K = 2 and K = 3, which had similar deltaK values, indicated panmixia (Fig. 3; Additional file 4: Fig. S1).

Kinship analyses (SEQUOIA) revealed approximately 45% of individuals were related (36 probable relationships among the 81 samples), including parent-offspring (PO = 2), full siblings (FS = 3), grandparent (GP = 29) and half avuncular (HA = 2) - great-grandparents/cousins (Additional file 2: Table S2), among BCS, NCS, and CTS. The relationships predicted are indicative of gene flow among the sites and suggest BCS (shallowest and northernmost seep) serves as a source population, supplying gametes/larvae to NCS (deepest and southernmost seep). These analyses also indicate that at least 14 out of 81 samples (17%) at NCS are sourced from NCS, suggesting high local recruitment.

**Table 5:** Inferred (posterior mean) migration rates calculated using neutral loci

	**Source**			
**Target**	**SITE**	*NCS*	*CTS*	*BCS*
	*NCS*	**0.7440**	0.0060	0.2500
	*CTS*	0.0352	**0.7678**	0.1969
	*BCS*	0.0666	0.0134	**0.9200**

Values represent the proportion of individuals from each site (row) that are either non-migrants (bold) or migrants derived from another site. Migrant sources are listed at the top of the table. NCS= Norfolk Canyon Seep, CTS= Chincoteague Seep, BCS= Baltimore Canyon Seep

Further assessments of gene flow (BA3) between the three seep sites (NCS, CTS, BCS) dominated by *G. childressi* indicated some degree of gene flow in this system (Table 5). At least 25% of individuals at NCS were migrants from BCS. Similarly, approximately 20% of migrants at CTS were from BCS. However, BayesAss also indicated a high fraction of non-migrants at each seep site (75–92%) (Table 5).

#### *Bathymodiolus heckerae*

On average ~ 99.9% of reads for *B. heckerae* passed the additional quality filtering step in iPYRAD, which generated an average of ~ 98.4 K total clusters per individual with a heterozygosity estimate of 0.01 and an error estimate of 0.003. Following assembly, there were 4142 filtered (max. 20% missing data) loci remaining of the 259,443 assembled in total using the *G. platifrons* genome, yielding 52,904 total SNPs (37.31% missing sites and 4114 unlinked SNPs).

Assessments of site-wide genetic differentiation among *B. heckerae* individuals collected at BRS yielded an overall* F*_*ST*_ of 0.00 and an inbreeding coefficient (*F*_*IS*_) of approximately 0.30 (Table 2), similar to the high-level of inbreeding found among *G. childressi*. The low overall mean observed heterozygosity (*H*_*O*_ = 0.07), within population gene diversity (*H*_*S*_ = 0.10) and overall gene diversity (*H*_*T*_ = 0.10) also indicate low heterozygosity and relatively equal allele frequencies among *B. heckerae* individuals collected in the Blake Ridge region.

**Fig. 4:**
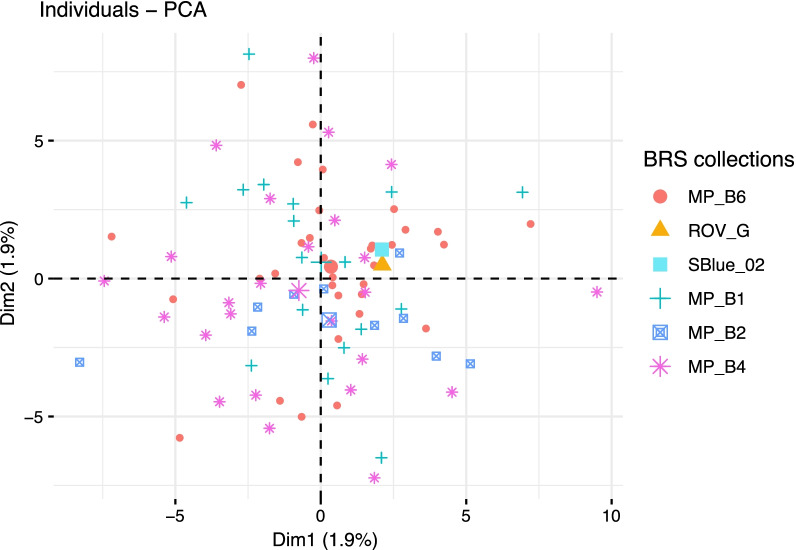
Principal Components Analysis (PCA) plot representing genetic differentiation among *B. heckerae* samples from six discrete collections along Blake Ridge Seep (BRS) (1, red circle: mussel pot B6 [n = 33], 2, orange triangle: remotely operated vehicle grab- ROV_G [n = 1], 3, filled blue square: slurp- SBlue_02 [n = 1], 4, teal cross: mussel pot B1 [n = 17], 5, blue square: mussel pot B2 [n = 10], 6, pink asterisk: mussel pot B4 [n = 25])

To identify whether there was cohort-level population structure among *B. heckerae* a PCA was performed to visualize genetic differentiation among *B. heckerae* individuals—with samples grouped by collection (1: mussel pot B6 [n = 33], 2: ROV_G [n = 1], 3: SBlue_02 [n = 1], 4: mussel pot B1 [n = 17], 5: mussel pot B2 [n = 10], 6: mussel pot B4 [n = 25]). This revealed broad genetic similarity among the discrete collections via a considerable amount of overlap and a lack of distinct clustering by collection group (Fig. 4).

The kinship analyses (SEQUOIA) revealed approximately 10% of the sampled *B. heckerae* individuals were related (Eight probable relationships among the 87 samples), including grandparent (GP = 6) and half avuncular (HA = 2)—great-grandparental/ cousins (Additional file 3: Table S3). This suggests relatively high levels of local recruitment despite large dispersal capabilities, considering the subsample of individuals examined here from Blake Ridge seep. These kinship results also included a probable grandparental relationship between sample *HRS-1704-CM-35* collected in a region near Norfolk Canyon, and individual *CM-00151* collected with mussel pot B6 at BRS, indicating relatively recent dispersal and highlighting gene flow capabilities similar to *G. childressi.*

### Signatures of selection

**Fig. 5:**
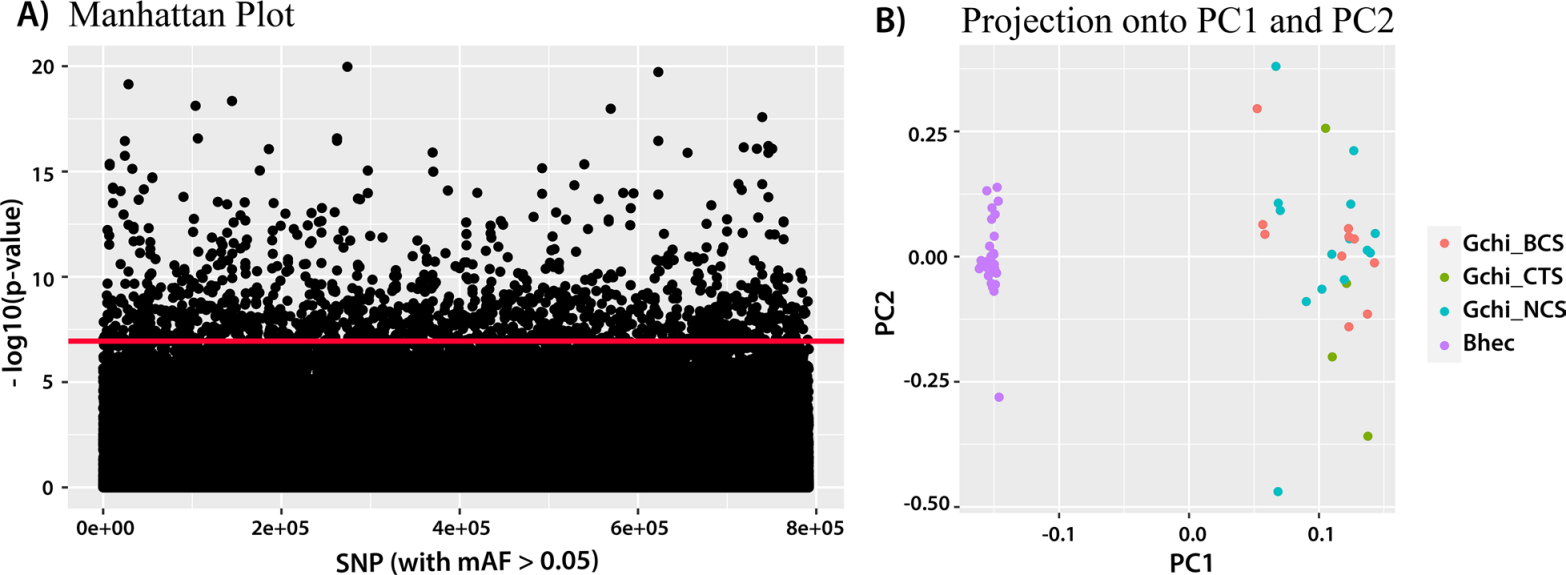
**A:** Manhattan plot revealing outlier SNPs (adjusted p < 0.05, above red line) with a minimum allele frequency (mAF) > 0.05 (default). **B:** A score plot displaying the projection of each sample onto the principal components (PC) of the PCA conducted in *pcadapt*. Samples are color coded by site for *G. childressi* (Baltimore canyon seep (BCS), pink); Norfolk Canyon seep (NCS), blue; and Chincoteague seep (CTS), green) and/or species *B. heckerae* (purple)

Using conservative methods for outlier detection between the two bathymodioline species, 3,429 outlier SNPs (both linked and unlinked) were identified using an adjusted p-value cutoff of 0.05 (Fig. 5A). The score plot from pcadapt (Fig. 5B), which displays the projection of each sample onto the principal components of the PCA, revealed species structure among the SNP outliers; *B. heckerae* specimen HRS-1704-CM-35 from the NCS region was distinct from the *B. heckerae* from BRS. PCA analysis also indicated no clustering of *G. childressi* sampled at the different canyon sites. These data suggest that there is no evidence for adaptation with gene flow between BCS, NCS and CTS. Analysis of SNP outliers (n = 3,429) specific to *G. childressi* also confirmed a lack of population structure among the sampled SNPs (Additional file 5: Fig. S2).

**Fig. 6:**
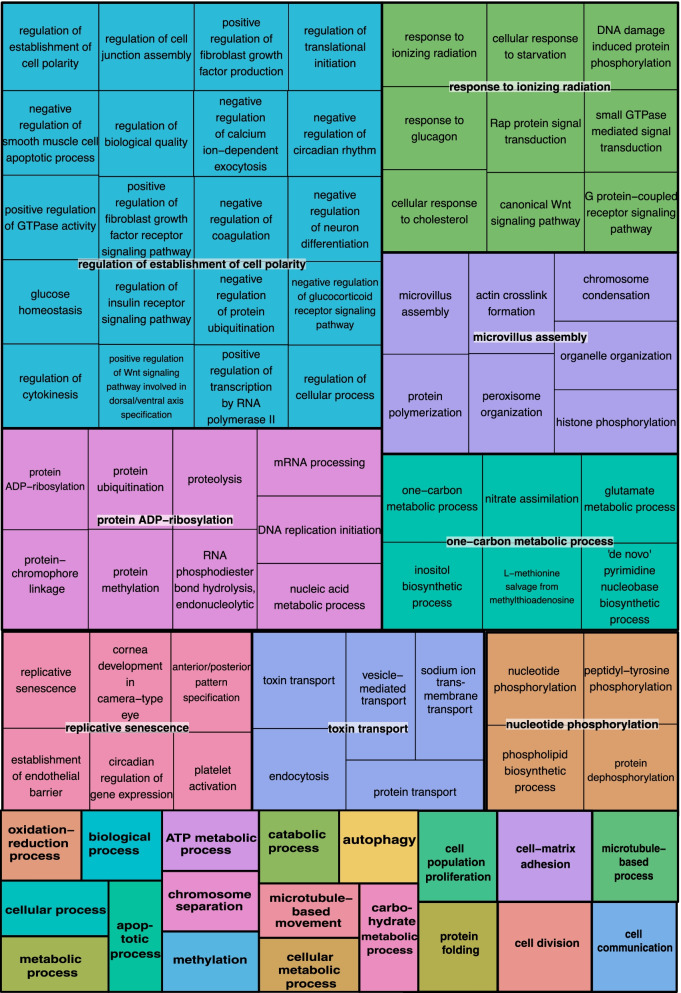
REViGO treemap summary of Gene Ontology information for biological processes associated with the outlier SNPs in the two bathymodioline species. Color blocks represent grouped terms based on common overlapping ontology

Based on the annotations for the *G. platifrons* genome, we obtained annotation information for 1,259 of the outlier SNPs, which corresponded to 427 unique *G. platifrons* genes (Additional file 7: Table S6). Among those annotated genes are a variety of known environmental response genes including- a probable cytochrome p450, ABC transporters, collagens, various zinc finger proteins, solute carriers, serine-threonine kinases and ion receptors, glutathione S-transferase (GST), heat shock protein 70, carbonic anhydrases and the stress response protein nhaX, and a diagnostic cancer biomarker protein Cubillin. The gene list also included development-related genes, such as the developmental homeobox gene Hox-A9, and the transcription factor SOX-30 involved in Wnt signaling and spermatid development. Additionally, sulfite oxidase was an outlier, which is a gene involved in sulfur metabolism. Complementary Gene Ontology (GO) analyses also indicated that the loci under putative selection in bathymodiolines are associated with a variety of biological processes including ATP and carbohydrate metabolism, response to ionizing radiation (i.e., reactive oxygen species (ROS) and DNA damage), inhibition of coagulation, toxin transport, microtubule-based movement, protein folding, epigenetic modifications (such as methylation, phosphorylation and ubiquitination), cellular response to starvation, nitrate assimilation, Wnt signaling, establishment of an endothelial barrier, and organelle and cytoskeletal organization (Fig. 6). Grouping these GO terms based on overlapping ontology terms revealed various putative adaptive loci function in responding to environmental stress (e.g. response to ionizing radiation, toxin transport), cellular regulation and organization, DNA and protein modifications and metabolism (Fig. 6, Additional file 7: Fig. S3).

BRITE functional hierarchy of the KEGG annotations retrieved from the *G. platifrons* genome indicated that the SNPs correspond to loci associated with three primary protein families- (1) metabolism, (2) genetic information processing, and (3) signaling and cellular processes (Additional file 6: Table S6). The most highly represented functional categories within those families include: (1: metabolism) enzymes (n = 67), protein kinases, phosphatases and associated proteins (n = 16), (2: genetic information processing) chromosome and associated proteins (n = 30), membrane trafficking (n = 16), ubiquitin system (n = 11) and DNA repair and recombination proteins (n = 9), and (3: signaling/cellular processes) exosome (n = 14), transporters (n = 8) and cytoskeletal proteins (n = 8). KEGG pathway reconstruction further revealed functional associations with, but not limited to, microbial metabolism in diverse environments, sulfur and nitrogen energy metabolism, regulation of actin cytoskeleton, circadian rhythm and thermogenesis (Additional file 6: Table S6).

## Discussion

### Genetic connectivity and diversity

*Gigantidas childressi* is a chemosynthetic-ecosystem engineer distributed widely throughout the North Atlantic Ocean. To date, this species has been recorded from cold seeps and brine pools in the Gulf of Mexico [56], along the Mid-Atlantic U.S. margin [13, 50], and off Trinidad and Tobago [57]. This widespread broadcast spawning species, with a long (up to 16.5 months) planktotrophic dispersal phase and vertically migrating larvae [58–61], has the ability to disperse long distances across a range of environmental conditions and thus colonize a variety of chemosynthetic habitats in the deep sea [62]. Similarly, *Bathymodiolus heckerae* is distributed widely throughout the North Atlantic, with records in the Gulf of Mexico [56, 63], Mid-Atlantic margin [12], and Blake Ridge off South Carolina [3]. Based on distribution patterns and life history characteristics, it is not too surprising that population structure was not detected among *G. childressi* sites separated by 135 km and > 1000 m difference in depth or among collections of *B. heckerae* at Blake Ridge. Both STRUCTURE and PCA plots combined with very low to negligible F_ST_ values indicated a high degree of shared genetic variation among *G. childressi* from the different sites, indicating that there is likely one large population of *G. childressi* present across the Mid-Atlantic region; additional sampling efforts would confirm the extent of this connectivity. Our results are similar to findings for *G. childressi* populations in the Gulf of Mexico, which showed minimal genetic differentiation over 500 km of geographical distance and 1500 m vertical distance based on restriction fragment length polymorphism data [16, 64].

We were able to estimate contemporary gene flow rates and directionality among sites with *G. childressi* sub-populations. BayesAss analysis, which can infer gene flow rates and directionality over the last few generations [48], indicated that ~ 25% of migrants moved from the shallower BCS site to the deeper NCS site and ~ 20% moved from BCS to the intermediate CTS site. A relatively negligible fraction (8%) of migrants from NCS and CTS were found at BCS. In addition, the kinship analyses suggested that many of the kin relationships observed were sourced from BCS. This pattern is consistent with an onshore to offshore or down slope pattern of dispersal, which has been hypothesized to be a predominant mode of genetic diversification in deep-sea taxa (e.g., [65–67]). However, this directionality in gene flow, from BCS to CTS and NCS, is also oriented in a north to south direction, which corresponds with the prevailing current structure in the region. The Labrador Current (and derived Labrador Slope water) is the predominant current along the shelf and slope off the northeastern U.S., bringing cold waters from the northern Labrador Sea southward to an area off Cape Hatteras, North Carolina, where the Labrador Current converges with the Gulf Stream [68, 69]. The high level of non-migrants (92%) calculated at BCS is also noteworthy, but we expect this estimate is because source populations north of BCS were not included in this study. Nevertheless, our results indicate that BCS is an important source of genetic material to downstream and deeper sites in the region.

Unexpectedly, kinship analyses suggested a high degree of relatedness among individuals, not only among sites but also within a particular site. Of the *G. childressi* individuals collected from the three seep sites, 45% were kin (and total inbreeding coefficient among the sites was high). Notably, 17% of *G. childressi* were related at the NCS. Similarly, 10% of *B. heckerae* were related to one another at Blake Ridge, while a high inbreeding coefficient was estimated at this site as well. This was quite surprising as life history strategies of this species (i.e., long pelagic larval duration, vertical migration of larvae) suggest long-range dispersal. So, how do bathymodioline larvae disperse and then recruit back to natal or nearby natal sites?

We hypothesize that local current patterns serve as conduits for entrainment and dispersal of vertically-migrating [61] bathymodioline larvae. Just south of our study site off Cape Hatteras, North Carolina, the Gulf Stream separates from the continental margin and turns eastward across the North Atlantic Ocean. Downstream of this separation point, the Gulf Stream meanders off its mean path and sporadically impinges onto the study area, particularly over the NCS site [70]. As the Gulf Stream moves seaward, meanders grow in amplitude, leading to the formation of warm-core eddies that entrain shelf waters [70]. The eddies circle clockwise, last for 30–90 days [71] and propagate across the Mid-Atlantic U.S. margin. We hypothesize that Gulf Stream meanders and eddies could entrain bathymodioline larvae and advect them back to the Mid-Atlantic margin, enabling periodic local recruitment. Similarly, larval dispersal models for the glass sponge *Vasella pourtalesii* indicated low dispersal distances and high retention for a population simulated in this region [72]. Chemical cues from the chemosynthetic environment could subsequently trigger bathymodioline larvae to settle onto the benthos (see [73]). Larval entrainment and advection via Gulf Stream eddies are a well-known mechanism of larval fish transport in this region [74].

An alternative mechanism of retention could be associated with nearby submarine canyons. Submarine canyons have highly dynamic hydrography (e.g., [75, 76]). Benthic lander observations combined with conductivity-temperature-depth casts have indicated complex water mass structure, current speeds and directions, tidal forcing, and internal waves within the canyons [77]. Although the exact mechanisms are unclear, it is possible that larvae are entrained within canyon environments within particular months or seasons [78]. *G. childressi* larvae are known to ontogenetically migrate to the surface, but they have also been collected near the seabed [58, 61, 73]. Therefore, it is possible that larvae can survive (weeks to months) in the canyon-rich environments and remain relatively close to their natal seep sites.

Notably, our kinship results also suggested that at least 10 and 17% of individuals were related at both NCS and BRS, respectively. These percentages seem quite high for a random sampling of a relatively small portion of the sub-population at each site. One hypothesis that has been proposed to explain high occurrences of kin in settlement patches is the ‘sweepstakes reproductive success’, whereby relatively few individuals produce a majority of successful recruits due to stochastic processes [79–81]. Variance in reproductive success could limit the effective population sizes, further reducing genetic diversity through genetic drift [80]. Alternatively, the observed patterns of relatedness could be driven by successful dispersal events of kin that remain in cohesive or collective dispersal kernels [82]. Sweepstakes reproductive success and/or a founder event of a cohesive larval cohort(s) of bathymodiolines could perhaps contribute to the high inbreeding coefficients and low observed heterozygosity within both bathymodioline species. We suggest that future research explore these hypotheses through further sampling and estimation of kin relationships across the spatial structure of bathymodioline mussel beds.

### Signatures of selection

Results of our study indicated that 427 genes are potentially under selection between the bathymodioline species surveyed. Based on the KEGG annotations, many of these genes can be grouped under three primary protein functional families: (1) metabolism, (2) genetic information processing, and (3) signaling and cellular processes. These genomic differences are likely related to environmental adaptations of the two species combined with the endosymbiont repertoire that each species harbors. In addition, some genes associated with development were found as outliers, suggesting potential developmental differences between these bathymodioline species. Below we highlight some of these genes under the three protein families, particularly as they relate to environmental or chemosynthetic adaptations, but we provide the full list of 427 genes in Additional file 6: Table S6. Our hope for the below discussion is that it will foster further investigation into gene function and evolution of bathymodiolines to advance our understanding of the biology and diversification of this successful group of chemosynthetic inhabitants.

Genes associated with metabolism were found to be under putative selection. Of particular interest here is the enzyme sulfite oxidase, which catalyzes sulfite to sulfate, and is known to play an important role in metabolism of sulfur-containing amino acids and in detoxifying exogenous sulfite and sulfur dioxide [83]. In cold seeps, reduced sulfur (i.e., hydrogen sulfide) can seep from the seafloor along with methane [84]. Reduced sulfur can be oxidized by free-living bacteria and via thiotrophic symbionts that live within the gill tissues of some bathymodioline mussel species (to help obtain nutrition, [12, 19, 84, 85]). Thus, sulfite oxidase is potentially under selection in bathymodiolines to fully utilize available sulfide sources, allowing them to thrive in sulfide-rich chemosynthetic habitats. A recent gene expression study on the bathymodioline *G. platifrons* indicated that several genes in the sulfide oxidation pathway, including sulfite oxidase, are upregulated in response to increased sulfide concentrations [99]. Although recent studies indicated that some individuals of *G. childressi* from BCS and NCS can harbor thiotrophs [12] and 14–16% of their nutrition can be derived from hydrogen sulfide [86], *B. heckerae* is known to harbor multiple thiotroph phylotypes [18] and potentially derive up to 40% of their carbon (at BRS) from thiotrophic symbionts [3]. Thus, selection of sulfite oxidase may be driven by endosymbiont repertoire. If both mussel species are capable of benefiting from thiotrophy, the lack of (large-scale) co-occurrence among *B. heckerae* and *G. childressi* may be associated with competition for limited resources, in which *B. heckerae*’s diverse repertoire of endosymbionts may enable them to outcompete other seep bivalves. It is also possible that selection of sulfite oxidase is driven by high concentrations of reduced sulfur in the environment. Further investigation is needed to elucidate the differences in seepage conditions between the ridge (deeper) and canyon (shallower) seeps.

Our candidate SNPs associated with gene sets enriched for functions related to genetic information processing were found to be outliers and potentially under selection. One functional gene category, the ubiquitin system (here including ubiquitin activating enzymes, regulators and ubiquitin-protein ligases), is known to be important in response to environmental stress [87, 88]. This system is involved in rapid degradation of short-lived proteins [87] and plays a critical role in regulation of physiological processes in both animals and plants [88]. In plants, for example, the ubiquitin system has been shown to be important for tolerance to drought, salinity, cold, nutrient deprivation and pathogens, enabling them to efficiently respond to environmental stress [88]. Marine invertebrates up-regulate ubiquitin in response to temperature extremes (see [89]). Spatial and temporal variation in protein ubiquitination has been observed for the mussel *Mytilus galloprovincialis* and associated with cold stress and increased protein turnover. This is in contrast to congeners that are less sensitive to cold temperatures [90]. We hypothesize that this system is under selection for temperature-related adaptation, as *B. heckerae* is generally found in much deeper depths than *G. childressi* and thus subjected to colder temperatures. Ubiquitin proteins have been found to be under positive selection in deep-sea hydrothermal vent crustaceans [91] and differentially expressed in marine bivalves in response to other environmental stressors (e.g., trace metals [92]). Alternatively, putative selection in the bathymodioline ubiquitin system could be related to regulating symbioses, as it has been shown that the ubiquitin system can be up-regulated in response to experimental symbiont loss in *Bathymodiolus* [93].

Signaling and cellular processing genes associated with candidate SNPs, including eight cytoskeletal proteins, were also overrepresented in our SNP outlier analyses. Similarly, in a study of the deep-sea fish, *Aldrovandia affinis*, a set of proteins involved in cytoskeleton organization (particularly proteins stabilizing actin and microtubules and nucleic-binding proteins involved in genetic information processing) had clear signatures of positive selection [94]. We hypothesize that cytoskeletal proteins are under positive selection in *B. heckerae* to tolerate extreme environmental conditions, such as sulfide-rich environments, cold temperatures and high hydrostatic pressure. Cytoskeletal proteins are known to be pressure sensitive, with actin and tubulin dissociating under pressure (see [95]), and these proteins are often upregulated in gene-expression studies investigating environmental pollution, temperature and salinity stress in bivalves [96–98].

## Conclusions

With more than 20K SNPs recovered from each bathymodioline species, we provided the first estimates of genome-wide genetic diversity of these deep-sea ecosystem engineers along the U.S. Atlantic margin. Our analyses indicated lack of population differentiation within *G. childressi* among three sites spanning 135 km and > 1000 m depth. Further, our analyses indicated that contemporary gene flow within *G. childressi* occurs from shallow to deeper depths in a southerly direction, indicative of an onshore to offshore pattern of connectivity that is also consistent with the directionality of the southward-flowing Labrador Current. Our unique kinship analyses further supported these conclusions and highlighted that 10–20% of individuals at any one site are kin; raising the question of how these highly interconnected populations, with high dispersal rates, are also able to locally recruit to a site or a nearby site from which they were spawned. Finally, signatures of putative selection associated with 427 genes provided clues into species-specific adaptations that could enable survival, persistence, and potential speciation within chemosynthetic ecosystems in the deep sea. We urge further research on these functional categories of genes, including, metabolism, genetic information processing, and signaling and cellular processes, which could significantly advance our understanding of the biology and evolution of this successful group of chemosynthetic inhabitants.

## Supplementary information

**Additional file 1. Table S1.** Sample information for Gigantidas childressi and Bathymodiolus heckerae individuals collected from the study area from either Norfolk Canyon Seep (NCS), Baltimore Canyons Seep (BCS), Chincoteague Seep (CTS) or Blake Ridge Seep (BRS). The asterisk (*) denotes samples used in selection analyses, while the (x) denotes samples excluded from analyses based on sequencing results.

**Additional file 2. Table S2**. Kinship associations and associated log-likelihood ratios (LLR) among G. childressi individuals (ID) collected from the three different seep sites as predicted by SEQUOIA. TopRel = second column ID relative to first column ID and includes parent-offspring (PO), full siblings (FS), grandparent (GP) and half avuncular (HA) - great-grandparents /cousins.

**Additional file 3. Table S3**. Kinship associations and associated log-likelihood ratios (LLR) among B. heckerae individuals (ID) collected from Blake Ridge seep, with one individual collected near Norfolk canyon, as predicted by SEQUOIA. TopRel = second column ID relative to first column ID and includes grandparent (GP) and half avuncular (HA) - great-grandparents/cousins.

**Additional file 4. Figure S1.**. Average probability of membership graph for G. childressi (n = 81) collected from seeps at Norfolk canyon (NCS), Chincoteague (CTS) and Baltimore Canyon (BCS). K = 3 clusters (ancestral populations), as identified by STRUCTURE, and also reported here as it had similar deltaK values as K=2, both indicating panmixia.

**Additional file 5. Figure S2.** A score plot displaying the projection of each G. childressi sample, used in the selection analyses, onto the principal components of the PCA conducted in pcadapt. Samples are color coded by seep site: Baltimore Canyon Seep (BCS), pink; Norfolk Canyon Seep (NCS), blue; and Chincoteague Seep (CTS), green.

**Additional file 6. Table 6.** Annotation information extracted from the G. platifrons genome (NCBI accession GCA_002080005.1 [36]), for the 427 unique bathymodioline genes corresponding to the outlier SNPs detected with pcadapt.

**Additional file 7. Figure S3.** Reduced representation of the Gene Ontology (GO) Biological Process categories shown in Figure 6, and corresponding putative number of genes linked to each process, that are associated with the outlier SNPs identified with pcadapt.

## Data Availability

The datasets supporting the conclusions of this article are available in the Sequence Read Archive (SRA) database under BioProject PRJNA769076. The COI barcode dataset generated for *B. heckerae* are available on Genbank (TBD). The COI barcode dataset used for *G. childressi* are publicly available on NCBI (PopSet: 1,485,777,102). Code to accompany the RADseq data processing, assembly and analyses can be found on GitHub (https://github.com/deleod).

## Abbreviations

RADSeq: Restriction-Site Associated DNA Sequencing
SNPs: Single nucleotide polymorphisms
NCS: Norfolk Canyon Seep
BCS: Baltimore Canyon Seep
CTS: Chincoteague Seep
BRS: Blake Ridge Seep
HOV: Human-operated vehicle
ROV: Remotely-operated vehicle
HWE: Hardy-Weinberg Equilibrium
PCA: Principal Component Analysis
PC: Principal components
PO: Parent-offspring
FS: Full siblings
HS: Half siblings
GP: Grandparents
FA: Full avuncular (niece/nephew or aunt/uncle)
HA: Half avuncular (great-grandparental/cousins)
U: Unrelated
LLR: (log10) likelihood ratio
BA3: BayesAss v3.04 program
KO: KEGG orthology
