# Supplementary material for: Genetic diversity and connectivity of chemosynthetic cold seep mussels from the U.S. Atlantic margin
Source: BMC Ecol Evol. 2022 Jun 17;22:76. doi: 10.1186/s12862-022-02027-4 (PMC9204967; doi:10.1186/s12862-022-02027-4)
Supplement: Supplementary file 2 — Additional file 2. Table S2. Kinship associations and associated log-likelihood ratios (LLR) among G. childressi individuals (ID) collected from the three different seep sites as predicted by SEQUOIA. TopRel = second column ID relative to first column ID and includes parent-offspring (PO), full siblings (FS), grandparent (GP) and half avuncular (HA) - great-grandparents /cousins. [file 12862_2022_2027_MOESM2_ESM.pdf]

Table S2- Kinship associations and associated log-likelihood ratios (LLR) among *G. childressi* individuals (ID) collected from the three different seep sites as predicted by SEQUOIA. TopRel = second column ID relative to first column ID and includes parent-offspring (PO), full siblings (FS), grandparent (GP) and half avuncular (HA) - great-grandparents /cousins. NCS= Norfolk Canyon seep, CTS= Chincoteague seep, BCS= Baltimore Canyon seep.

ID1	ID2	TopRel	LLR	Site1	Site2
MAS339	**MAS541**	PO	0.72	NCS	BCS
MAS321	**MAS544**	PO	0.08	NCS	BCS
MAS338	**MAS541**	FS	0.51	NCS	BCS
HRS-1704-CM-041	**MAS545**	FS	0.24	NCS	BCS
MAS298	**MAS557**	FS	0.1	NCS	BCS
HRS-1704-CM-069	**MAS322**	GP	7.83	CTS	NCS
HRS-1704-CM-069	**MAS297**	GP	6.05	CTS	NCS
MAS289	**MAS299**	GP	5.04	NCS	NCS
MAS341	**MAS556**	GP	4.58	NCS	BCS
MAS293	**MAS544**	GP	4.01	NCS	BCS
HRS-1704-CM-011	**MAS284**	GP	3.8	NCS	NCS
MAS291	**MAS561**	GP	3.76	NCS	BCS
HRS-1704-CM-011	**MAS288**	GP	3.32	NCS	NCS

HRS-1704-CM-009	**HRS-1704-CM-039**	GP	2.99	NCS	NCS
MAS291	**MAS556**	GP	2.95	NCS	BCS
MAS290	**MAS341**	GP	2.78	NCS	NCS
MAS555	**MAS557**	GP	2.58	BCS	BCS
MAS297	**MAS321**	GP	2.55	NCS	NCS
MAS290	**MAS327**	GP	2.33	NCS	NCS
HRS-1704-CM-031	**MAS553**	GP	2.22	NCS	BCS
HRS-1704-CM-009	**MAS298**	GP	2.15	NCS	NCS
MAS299	**MAS540**	GP	2.01	NCS	BCS
HRS-1704-CM-031	**HRS-1704-CM-041**	GP	1.94	NCS	NCS
HRS-1704-CM-031	**MAS545**	GP	1.89	NCS	BCS
MAS299	**MAS327**	GP	1.53	NCS	NCS
HRS-1704-CM-015	**MAS545**	GP	1.33	NCS	BCS
MAS322	**MAS340**	GP	1.3	NCS	NCS
HRS-1704-CM-011	**HRS-1704-CM-025**	GP	1.29	NCS	NCS
MAS284	**MAS544**	GP	1.17	NCS	BCS

MAS292	**MAS553**	GP	0.98	NCS	BCS
HRS-1704-CM-015	**MAS311**	GP	0.78	NCS	NCS
HRS-1704-CM-043	**MAS537**	GP	0.77	NCS	BCS
HRS-1704-CM-017	**MAS541**	GP	0.67	NCS	BCS
HRS-1704-CM-009	**MAS283**	GP	0.58	NCS	NCS
HRS-1704-CM-021	**MAS555**	HA	1.33	NCS	BCS
HRS-1704-CM-039	**HRS-1704-CM-069**	HA	1.13	NCS	CTS
