# Supplementary material for: Genetic diversity and connectivity of chemosynthetic cold seep mussels from the U.S. Atlantic margin
Source: BMC Ecol Evol. 2022 Jun 17;22:76. doi: 10.1186/s12862-022-02027-4 (PMC9204967; doi:10.1186/s12862-022-02027-4)
Supplement: Supplementary file 3 — Additional file 3. Table S3. Kinship associations and associated log-likelihood ratios (LLR) among B. heckerae individuals (ID) collected from Blake Ridge seep, with one individual collected near Norfolk canyon, as predicted by SEQUOIA. TopRel = second column ID relative to first column ID and includes grandparent (GP) and half avuncular (HA) - great-grandparents/cousins. [file 12862_2022_2027_MOESM3_ESM.pdf]

Table S3- Kinship associations and associated log-likelihood ratios (LLR) among *B. heckerae* individuals (ID) collected from Blake Ridge seep (BRS), with one individual collected near Norfolk canyon (NCS), as predicted by SEQUOIA. TopRel = second column ID relative to first column ID and includes grandparent (GP) and half avuncular (HA) - great-grandparents/cousins.

ID1	ID2	TopRel	LLR	Site
CM-00167	**RB-19-136**	GP	28.01	Blake Ridge
CM-00157	**RB-19-119**	GP	27.34	Blake Ridge
RB-19-125	**RB-19-154**	GP	14.26	Blake Ridge
RB-19-132	**RB-19-168**	GP	13.09	Blake Ridge
CM-00134	**CM-00157**	GP	9.48	Blake Ridge
CM-00151	**HRS-1704-CM-35**	GP	6.32	Blake Ridge / NCS
CM-00149	**RB-19-130**	HA	6.17	Blake Ridge
RB-19-157	**RB-19-171**	HA	0.67	Blake Ridge
