# Supplementary material for: Genetic diversity and connectivity of chemosynthetic cold seep mussels from the U.S. Atlantic margin
Source: BMC Ecol Evol. 2022 Jun 17;22:76. doi: 10.1186/s12862-022-02027-4 (PMC9204967; doi:10.1186/s12862-022-02027-4)

Figure S1. Average probability of membership graph for *G. childressi* (n = 81) collected from seeps at Norfolk canyon (NCS), Chincoteague (CTS) and Baltimore Canyon (BCS). K = 3 clusters (ancestral populations), as identified by STRUCTURE, and also reported here as it had similar deltaK values as K=2, both indicating panmixia.

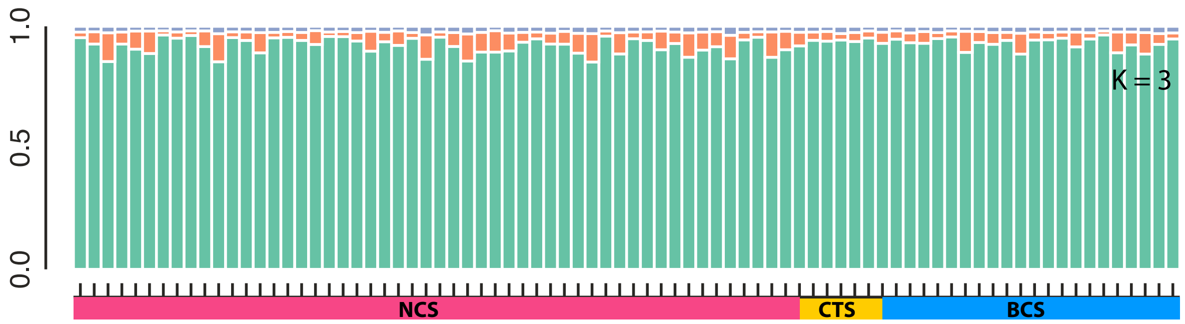

Supplement: Supplementary file 4 — Additional file 4. Figure S1.. Average probability of membership graph for G. childressi (n = 81) collected from seeps at Norfolk canyon (NCS), Chincoteague (CTS) and Baltimore Canyon (BCS). K = 3 clusters (ancestral populations), as identified by STRUCTURE, and also reported here as it had similar deltaK values as K=2, both indicating panmixia. [file 12862_2022_2027_MOESM4_ESM.pdf]
