# Supplementary material for: Genetic diversity and connectivity of chemosynthetic cold seep mussels from the U.S. Atlantic margin
Source: BMC Ecol Evol. 2022 Jun 17;22:76. doi: 10.1186/s12862-022-02027-4 (PMC9204967; doi:10.1186/s12862-022-02027-4)

Figure S2- A score plot displaying the projection of each *G. childressi* sample, used in the selection analyses, onto the principal components of the PCA conducted in *pcadapt*. Samples are color coded by seep site: Baltimore Canyon Seep (BCS), pink; Norfolk Canyon Seep (NCS), blue; and Chincoteague Seep (CTS), green.

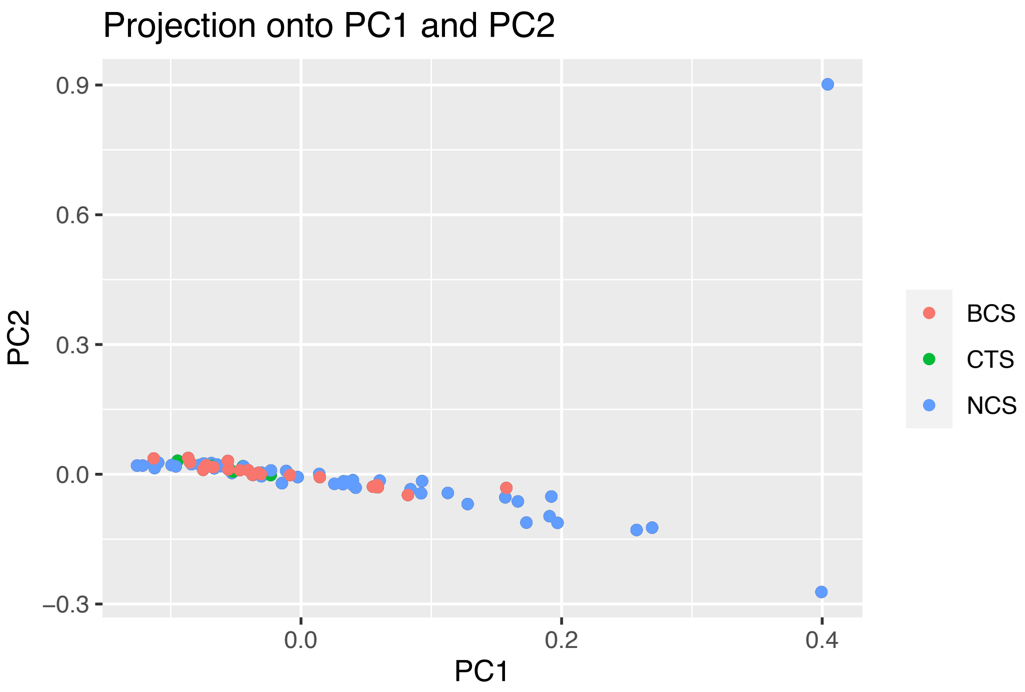

Supplement: Supplementary file 5 — Additional file 5. Figure S2. A score plot displaying the projection of each G. childressi sample, used in the selection analyses, onto the principal components of the PCA conducted in pcadapt. Samples are color coded by seep site: Baltimore Canyon Seep (BCS), pink; Norfolk Canyon Seep (NCS), blue; and Chincoteague Seep (CTS), green. [file 12862_2022_2027_MOESM5_ESM.pdf]
