# Supplementary material for: Genetic diversity and connectivity of chemosynthetic cold seep mussels from the U.S. Atlantic margin
Source: BMC Ecol Evol. 2022 Jun 17;22:76. doi: 10.1186/s12862-022-02027-4 (PMC9204967; doi:10.1186/s12862-022-02027-4)

Figure S3- Reduced representation of the Gene Ontology (GO) Biological Process categories shown in Figure 6, and corresponding putative number of genes linked to each process, that are associated with the outlier SNPs identified with *pcadapt*.

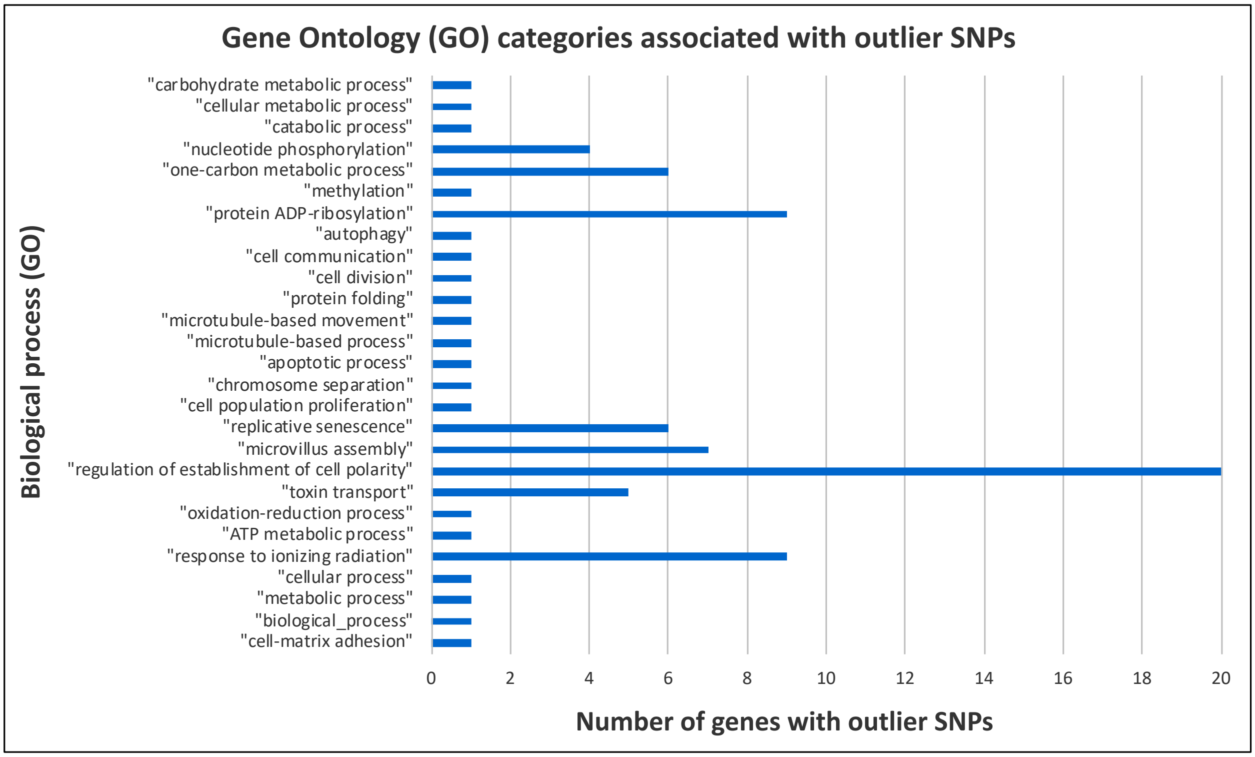

Supplement: Supplementary file 7 — Additional file 7. Figure S3. Reduced representation of the Gene Ontology (GO) Biological Process categories shown in Figure 6, and corresponding putative number of genes linked to each process, that are associated with the outlier SNPs identified with pcadapt. [file 12862_2022_2027_MOESM7_ESM.pdf]
